# MiR-199a-3p suppresses proliferation and invasion of prostate cancer cells by targeting Smad1

**DOI:** 10.18632/oncotarget.17191

**Published:** 2017-04-18

**Authors:** Feng Qu, Jinyu Zheng, Weidong Gan, Huibo Lian, Hua He, Wuping Li, Tian Yuan, Yaling Yang, Xiaogong Li, Changwei Ji, Xiang Yan, Linfeng Xu, Hongqian Guo

**Affiliations:** ^1^ Department of Urology, The Affiliated Nanjing Drum Tower Hospital, Medical School of Nanjing University, Nanjing, Jiangsu, 210008, China; ^2^ Institute of Urology, Nanjing University, Nanjing, Jiangsu, 210093, China; ^3^ Department of Pathology, The Affiliated Nanjing Drum Tower Hospital, Medical School of Nanjing University, Nanjing, Jiangsu, 210008, China; ^4^ Department of Hematopathology, Division of Pathology and Laboratory Medicine, University of Texas MD Anderson Cancer Center, Houston, Texas, 77030, USA; ^5^ Department of Lymphoma, Jiangxi Cancer Hospital, Nanchang, Jiangxi, 330029, China

**Keywords:** MiR-199a-3p, Smad1, prostate cancer, proliferation, invasion

## Abstract

**Objectives:**

This study was intended to analyze effects of miR-199a-3p and Smad1 on proliferation, migration and invasion of prostate cancer (PCa) cells.

**Results:**

MiR-199a-3p was significantly decreased in PCa tissues in comparison to that in adjacent normal tissues (*P* < 0.05). Over-expressed miR-199a-3p markedly suppressed proliferation and invasion of PCa cells (*P* < 0.05). MiR-199a-3p was negatively correlated with Smad1 expression, and overexpression of Smad1 could antagonize the effects of miR-199a-3p on PCa cells.

**Materials and methods:**

The PCa tissues and their adjacent normal tissues were collected from 54 PCa patients. Expressions of miR-199a-3p and Smad1 mRNA in tissues and cells were evaluated with real-time quantitative polymerase chain reaction (RT-qPCR), and immunohistochemistry assay was used to detect Smad1 protein expressions. The target relationship between miR-199a-3p and Smad1 was assessed by luciferase reporter assay. The PCa cell lines (*i.e*. PC-3 cells) were transfected with miR-199a-3p mimics and Smad1-cDNA. MTT and Transwell assays were applied to detect proliferative, migratory and invasive abilities of PCa cells.

**Conclusions:**

MiR-199a-3p suppressed proliferation and invasion of PCa cells by targeting Smad1.

## INTRODUCTION

Prostate cancer (PCa) is the second major cause of cancer-related deaths among American males, and the proportion of it among all cancers achieves up to 43% [1, 2]. Although the survival rate of the clinically localized PCa patients has been increasing owing to treatments of surgery and radiotherapy, the recurrence rate of PCa within 5 years remains high at 25% [3, 4]. As a result, the molecular mechanisms underlying PCa development need to be further explored, which allows us to find novel therapeutic targets for PCa.

MicroRNAs (miRNAs), a kind of short non-coding RNAs that are approx 22–25nt long, can modify mRNA translations within carcinogenic pathways through binding to 3′-UTR region of the target mRNA [5–7]. Accumulating studies have also demonstrated remarkable associations of miRNAs expressions with occurrence and progression of PCa [8–11]. Specifically, it was indicated that miR-345 was differentially expressed between PCa and benign prostatic hypertrophy based on miRNA microarray analysis [12]. Wang *et al*. reported that serum miR-19 and miR-519c-5p levels can predict negative pathology of PCa patients [13]. In addition, miR-199b might impose inhibitive effects on PCa development since lower miR-199a expressions were observed with aggravation of PCa [14, 15].

Interestingly, miR-199a directly targeting Smad1 (Gene accession number: X69881) was demonstrated to modulate the activation of pancreatic stellate cells (PSCs) and PSC-induced pro-tumoral effects in pancreatic cancer [16]. Besides, Lin *et al*. also indicated that miR-199a is a BMP2 reactive miRNA which negatively regulated the differentiation of early chondrocyte by directly targeting Smad1 transcription factors [17]. In fact, Smad1 not only served as a substrate of mitogen-activated protein kinases (MAPKs), but also exerted a major role in transferring signals from bone morphogenetic proteins (BMPs), thereby inducing development of various disorders, including PCa, fibrosis, cardiovascular diseases and so on [18–22]. Furthermore, the ALK2/Smad1 pathway activated by endoglin was found to inhibit motility of PCa cells, and down-regulated Smad1 expressions due to target of miR-345 could inhibit growth, invasion and migration of human PCa cells [12, 23]. Thus, the synthetic effects of miR-199a and Smad1 were hypothesized to be correlated with PCa development.

However, this research pointed that whether miR-199a-3p might regulate PCa development by targeting Smad1 was still lacking up to now. In the present study, we conducted comprehensive research on the molecular effect of miR-199a-3p targeting Smad1 on the proliferation and invasion of PCa cells.

## RESULTS

### Expressions of miR-199a-3p and Smad1 in PCa tissues and PCa cell lines

**Figure 1:**
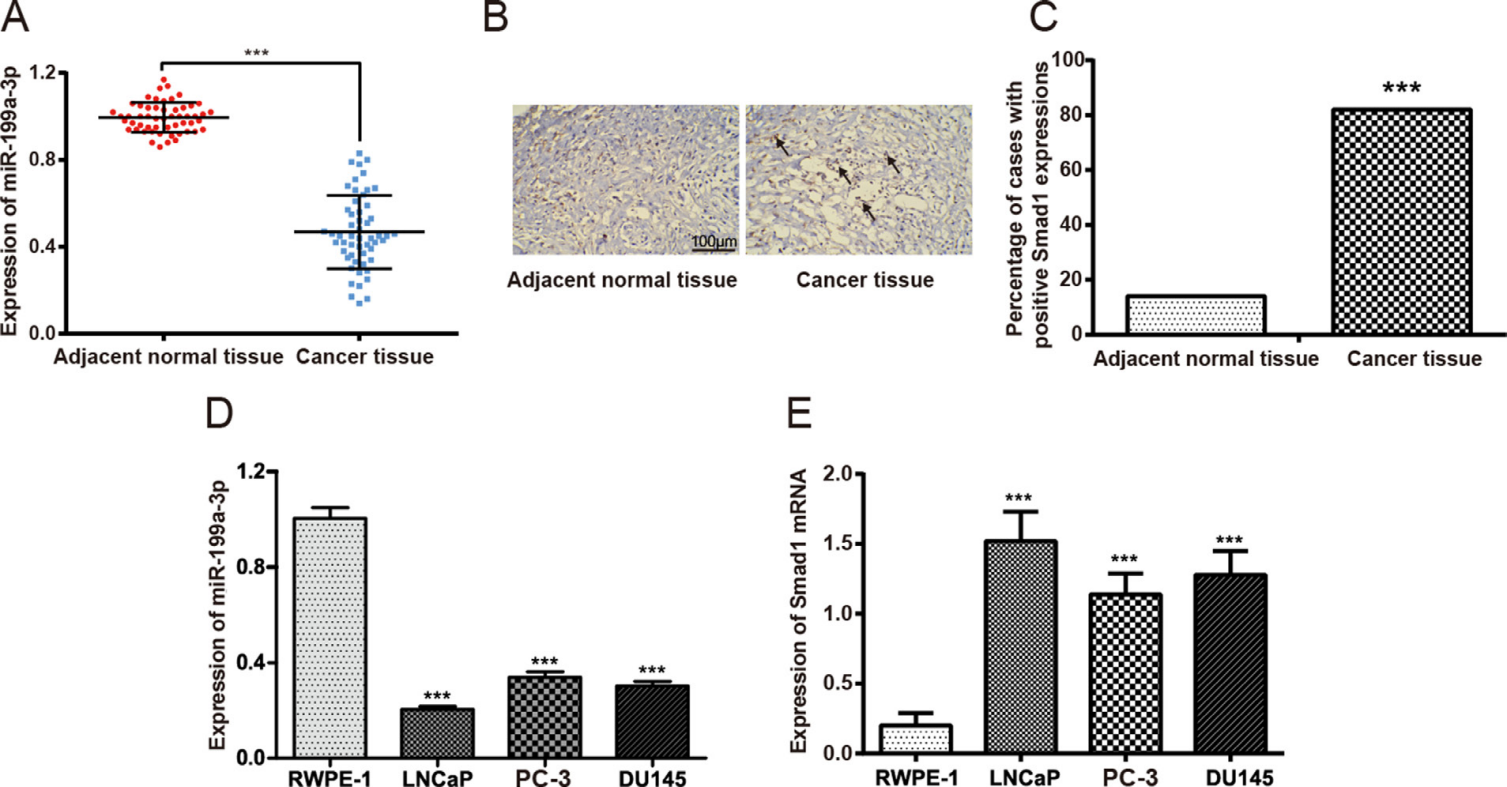
The expressions of miR-199a-3p and Smad1 in prostate tissue samples (**A**) Difference in the expression of miR-199a-3p between prostate cancer tissues and corresponding adjacent normal prostate tissues. Data were presented as mean ± SD. (**B**) Immunohistochemistry results showed the protein expression of Smad1 in prostate cancer tissues and cancer adjacent normal tissues. The red rectangles and black arrows have been pictured to direct Smad 1 positive staining (*i.e*. the brown colors). (**C**) Number of cases with positive Smad1 expressions. ***P < 0.001 compared with adjacent normal tissues. (**D**) The expression of miR-199a-3p in prostate cancer cell lines *(i.e*. LNCaP, PC-3 and DU145) and normal prostate epithelial cells (*i.e*. RWPE-1). ***P < 0.001 compared with RWPE-1. (**E**) The expression of Smad1 mRNA in prostate cancer cell lines (LNCaP, PC-3 and DU145) and normal prostate epithelial cells (*i.e*. RWPE-1). ***P < 0.001 compared with RWPE-1.

The expression of miR-199a-3p was significantly lower in PCa tissues than in adjacent normal tissues (*P* < 0.05) (Figure 1A). As displayed in Figure 1B–1C, the proportion of positively expressed Smad1 cells in PCa tissues (75.9%) significantly exceeded that in adjacent normal prostate tissues (12.9%). In addition, significantly down-regulated miR-199a-3p expressions and elevated Smad1 expressions were observed in PCa cell lines (*i.e*. LNCaP, PC-3 and DU145) in comparison to the normal prostate epithelial cell line (*i.e*. RWPE-1) (*P* < 0.05) (Figure 1D–1E). As PC-3 cells had higher expression of miR-199a-3p, it was used to perform subsequent cell experiments.

### MiR-199a-3p targeted to Smad1

**Figure 2:**
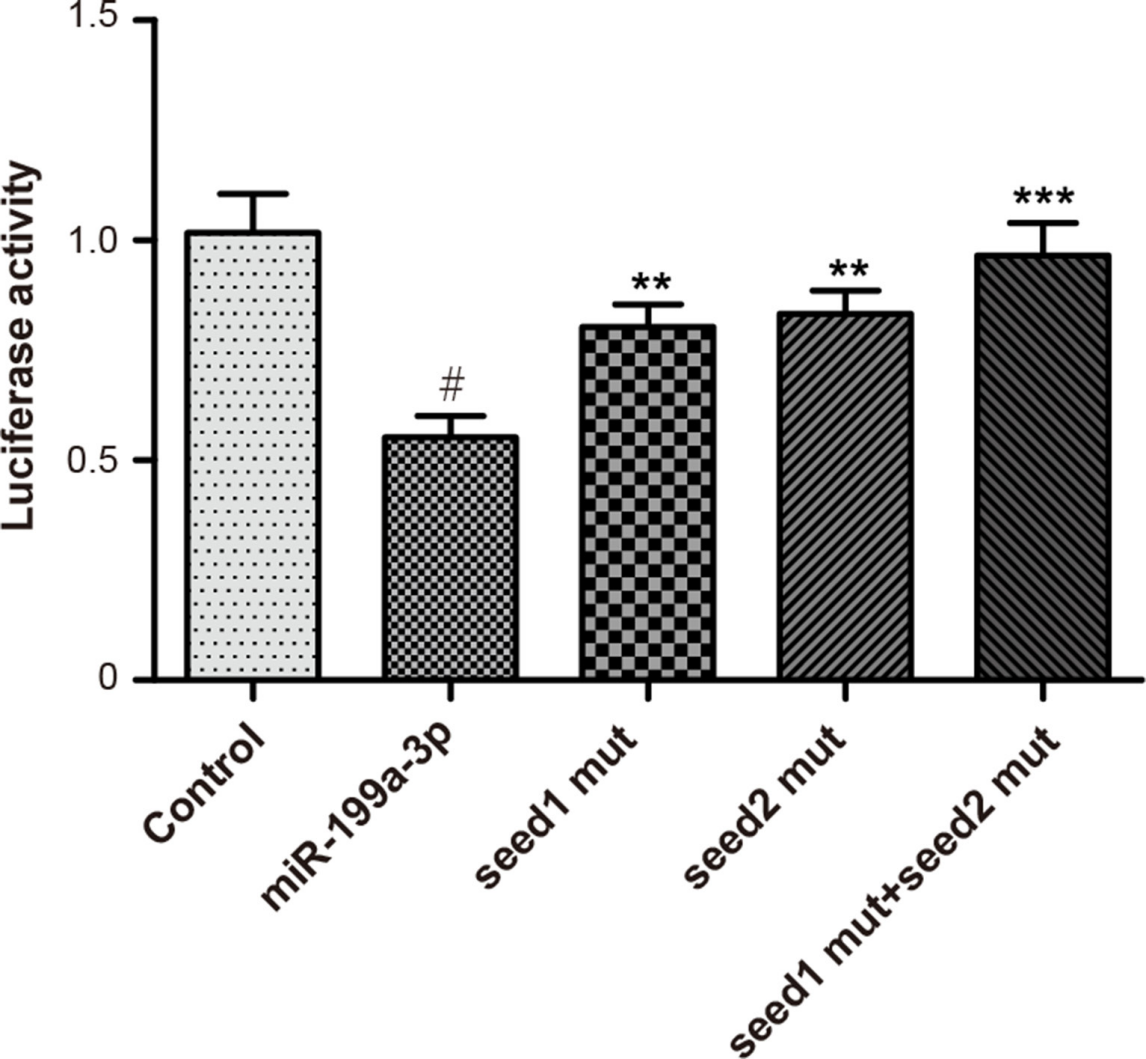
Luciferase activities were detected among groups of control, miR-199a-3p, Smad1 seed1 mut, Smad1 seed2 mut and Smad1 seed1+seed2 mut Data were presented as mean ± SD. ^#^*P* < 0.01 compared with control group; **P < 0.01 compared with miR-199a-3p group; ***P < 0.001 compared with miR-199a-3p group.

Perfect base pairing was observed between the sequence of mature miR-199a-3p and the 3′-UTR of Smad1 mRNA [17]. As suggested by luciferase reporter gene assay, miR-199a-3p group had a significantly lower relative light unit (RLU) value than the control group, whereas the RLU values of Smad1 seed1 mut group, Smad1 seed2 mut group, and Smad1 seed1 + seed2 mut group were significantly elevated in comparison to the miR-199a-3p group (*P* < 0.05) (Figure 2).

### MiR-199a-3p suppressed proliferation of PCa cells

**Figure 3:**
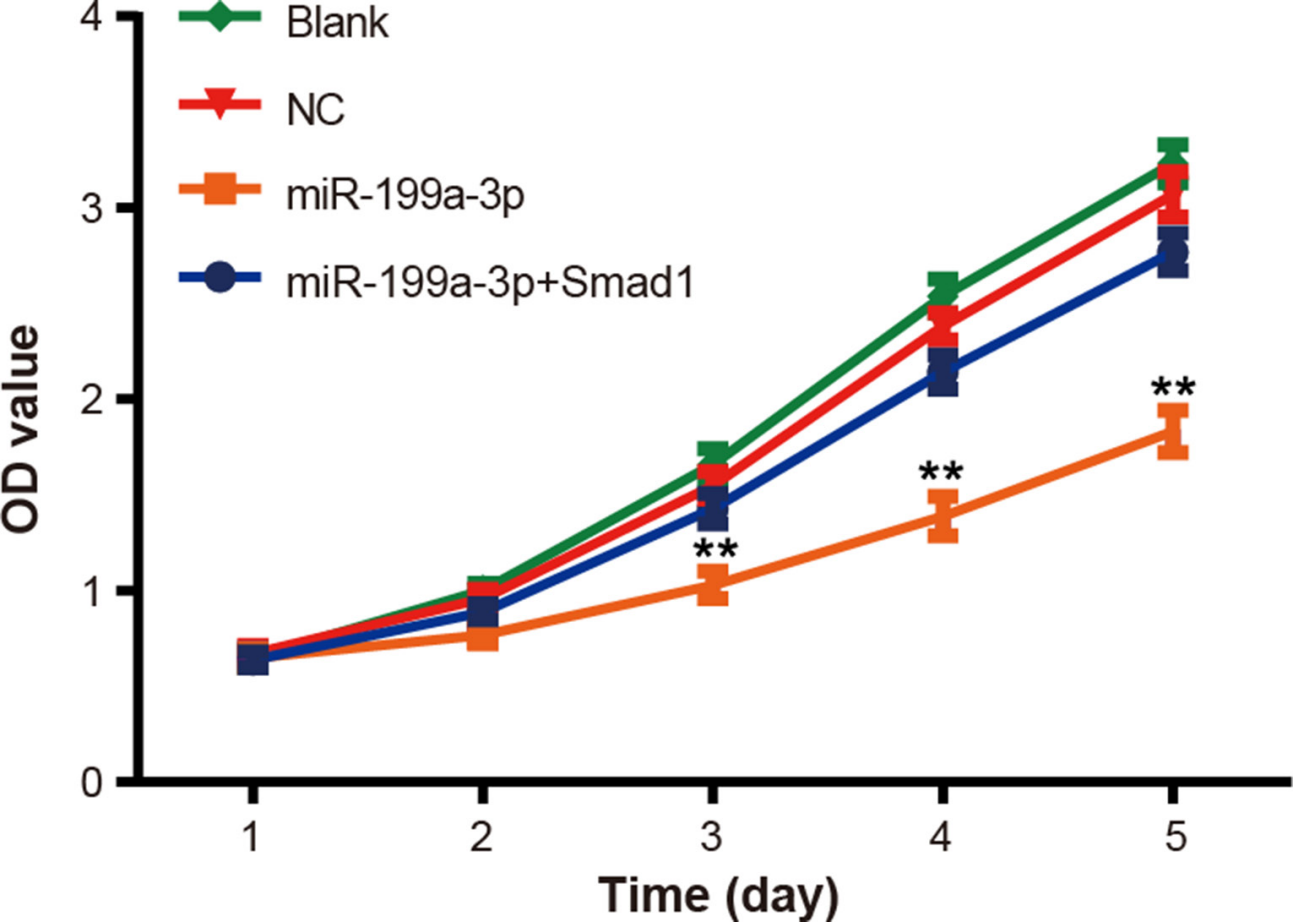
The effect of miR-199a-3p overexpression on PC-3 cell proliferation was evaluated by MTT assay Data were presented as mean ± SD. **P < 0.01 compared with NC group.

Significantly constrained proliferation of PCa cells transfected with miR-199a-3p mimics was observed when compared with the normal control group (*P* < 0.05) (Figure 3). Nonetheless, addition of Smad1 can reverse the inhibitory effects of miR-199a-3p on proliferation of PCa cells (*P* < 0.05). Moreover, no notable difference regarding motility of PCa cells was present among the blank group and normal control group at each time point (all *P* > 0.05).

### MiR-199a-3p inhibited migration and invasion of PCa cells

**Figure 4:**
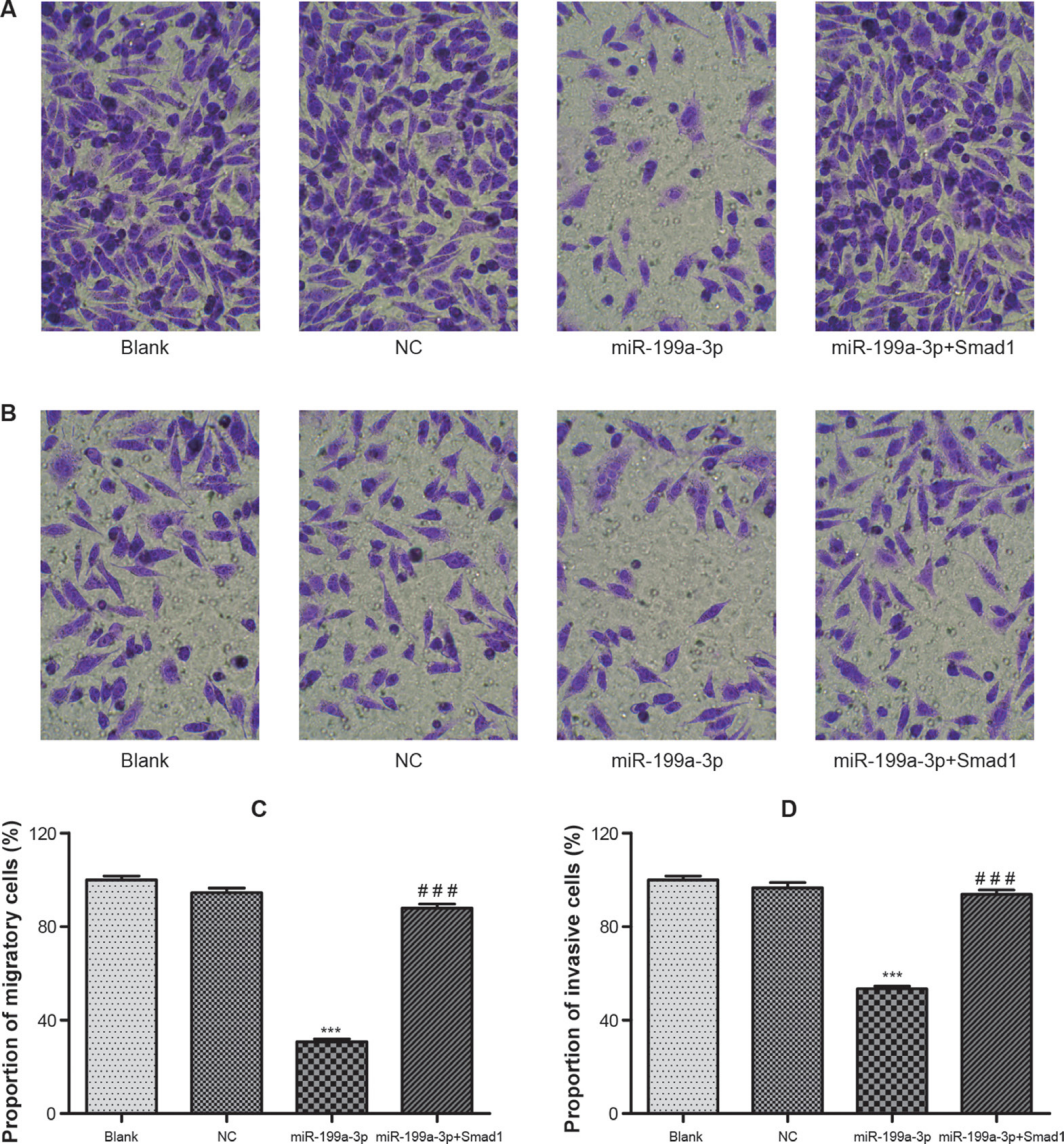
The migration and invasion of transfected PC-3 cells were detected by Transwell assay (× 200) (**A**) The migration of cells in groups of blank, NC, miR-199a-3p and miR-199a-3p+Smad1 was visualized under the microscope. (**B**) The invasion of cells in groups of blank, NC, miR-199a-3p and miR-199a-3p+Smad1 was visualized under the microscope. (**C**) Histogram of the migration rate of PC-3 cells. (**D**) Histogram of the invasion rate of PC-3 cells. Data were presented as mean ± SD. ***P < 0.001 compared with NC group; ^###^*P* < 0.001 compared with miR-199a-3p group.

The results of Transwell experiments indicated that up-regulation of Smad1 or down-regulation of miR-199a-3p could restrict the migratory and invasive ability of PC-3 cells (Figure 4A–4B). The average proportion of migratory cells in the miR-199a-3p mimics [(30.7 ± 2.15)%] group was significantly smaller than that in the normal control group (*P* < 0.05), while the normal control group [(94.5 ± 3.61)%] was nearly no different from blank control group (*P* > 0.05) (Figure 4C). As for invasion of PCa cells, the difference between the normal control group [(96.7 ± 3.92)%] and blank control group was insignificant (*P* > 0.05). However, the percentage of invasive cells in the miR-199a-3p mimics group [(53.4 ± 2.02)%] was significantly below that in the normal control group (*P* < 0.05) (Figure 4D).

### MiR-199a-3p suppressed Smad1 expression

**Figure 5:**
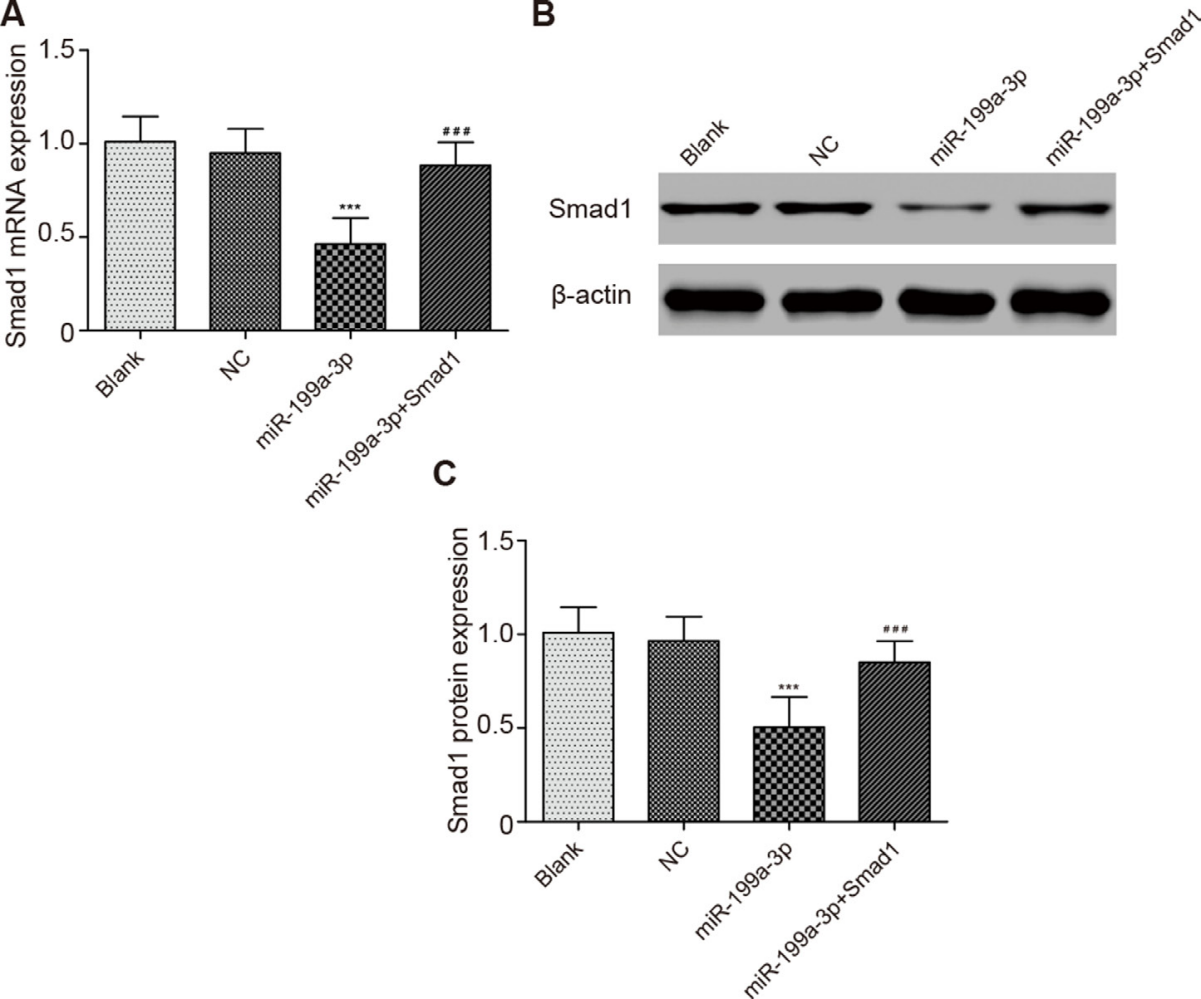
Comparison of Smad1 expressions in different groups (**A**) Comparison of Smad1 mRNA expression in different groups. (**B**) The effect of miR-199a-3p transfection on the PC-3 cells was detected by Western blotting assay. (**C**) Comparison of Smad1 protein expression in different groups. Data were presented as mean ± SD. ***P < 0.001 compared with NC group; ^###^*P* < 0.001 compared with miR-199a-3p group.

Scarcely any notable difference in Smad1 expressions was observed among the blank group, normal control group and miR-199a-3p + Smad1 group (*P* > 0.05). Furthermore, miR-199a-3p significantly down-regulated Smad1 expressions in comparison with the normal control group (*P* < 0.05), whereas the Smad1 expressions in the miR-199a-3p + Smad1 group was notably higher than those in the miR-199a-3p group (*P* < 0.05) (Figure 5).

## DISCUSSION

PCa now became a major public health problem within developed countries [24]. Its established risk factors mainly included aging, family history, hormones and genetic susceptibility [25, 26]. Accumulating evidences have indicated that miRNAs (*e.g*. miR-182, miR-130b and miR-345) may act as novel modulators for PCa development through regulation of mRNA of specific genes [*e.g*. *metalloproteinase-2* (*MMP-2*) and *Smad1*] [9, 11, 12].

Notably, miRNA-199a could trigger tumor progression through inhibiting functions of MMP9, a protein that could degrade the extracellular matrix [27]. Additionally, the reduction in miR-199a-3p expressions and thereby non-suppression of mammalian target of rapamycin (mTOR) could result in advanced forms of cancers [28]. A documentary also showed that miR-199a could serve to regulate metastasis of PCa cells through transforming growth factor-beta1 (TGF-β1)-dependant way [29]. Similarly, our study found that the expression level of miR-199a-3p was significantly lower in PCa tissues than that in adjacent normal tissues. We also discovered that expressions of miR-199a-3p in LNCaP, DU145and PC-3 cell lines were significantly decreased compared with human normal prostate epithelial cells. Besides, over-expressed miR-199a-3p suppressed the proliferation and invasion of PCa cells, implying that miR-199a-3p potentially played a restrained role in PCa development.

Smad proteins were recognized as central modulators of TGF-β and BMP signaling pathways, which modulated cell growth and differentiation [30, 31]. It was found that miR-155 decreased Smad1 and Smad5 expressions in the lung epithelial cell line A549, modulating the cyclin-dependent kinase suppressor p21 [32]. In addition, Liu *et al*. provided evidence that the Smad1/Akt/GSK3β pathway was related with Nanog expression induced by Snail and cancer stem cell-like transformation in non-small-cell lung cancer cells [33]. In our study, we found that the expression of Smad1 was significantly higher in PCa tissues than in adjacent normal tissues. Moreover, the expressions of Smad1 and Smad1 mRNA were both reduced after transfection of PC-3 cells with miR-199a-3p.

The targeting relationship between miR-199a-3p and Smad1 has also been emphasized to date, for instance, miR-199a-3p was found to negatively modulate differentiation of early chondrocyte by directly targeting Smad1 transcription factors [17]. Besides, it was documented that miR-199a-3p might modulate activation of pancreatic stellate cells (PSCs) and PSC-induced pro-tumoral effects by directly targeting Smad1 transcription factor [16]. In the present study, we used the luciferase reporter assay to confirm that Smad1 is a target gene of miR-199a-3p, and over-expression of Smad1 would antagonize the effects of miR-199a-3p on PCa cells. Therefore, our study revealed the potentially molecular mechanism of miR-199a-3p targeting Smad1 with respect to proliferation and invasion of PCa cells.

However, there still existed some limitations in our experiments. For example, only 54 pairs of clinical samples and one human PC cell line PC 3 were used to detect the influence of miR-199a-3p targeting Smad1 on tumor proliferation, migration and invasion. And it was necessary to study the relative molecular pathway, as miR-199a-3p had other targeting gene. Hence, the mechanism of miR-199a and Smad1 on development and progression of PCa needs to be studied further. In conclusion, miR-199a-3p exerts anti-growth, anti-invasion and anti-migration effects on human PCa by partially down-regulating Smad1, suggesting that miR-199a-3p might be a potential diagnostic and therapeutic target for patients with advanced PCa.

## MATERIALS AND METHODS

### Subjects

This study included 54 PCa patients which were treated with radical prostatectomy at the Affiliated Nanjing Drum Tower Hospital, Medical School of Nanjing University from March 2015 to September 2015. All patients were not treated with preoperative chemotherapy or radiotherapy treatment. All subjects underwent pathological diagnoses, and were confirmed to be PCa. The PCa tissues and corresponding adjacent normal tissues were frozen in liquid nitrogen and were preserved in −70°C refrigerator. Of note, the adjacent normal tissues were representative of tissues that were located about > 2 cm distant from tumors or in another lobe that was confirmed with no cancer cells. Biological biopsy has been performed to check that the adjacent normal tissues were not contaminated by PCa cells. All procedures were in accordance with the Declaration of Helsinki [34] and were approved by the ethics committee of Human Experimentation of the Affiliated Nanjing Drum Tower Hospital, Medical School of Nanjing University. All patients included in this study have signed consent forms.

### Immunohistochemistry for detecting expression of Smad1 protein

Smad1 protein expression was detected with PV-9000 two-step immunohistochemical method according to manufacturers’ instructions (ZSGB-BIO Cor., Beijing; catalog number: PV-9000). Specifically, conventional dewaxing and antigen microwave thermal retrieval were performed on four-micron-thick serial sections of formalin-fixed and paraffin-embedded tissues. The sections were incubated in 3% hydrogen peroxide to block endogenous peroxidase activity. Primary antibodies (Rabbit anti-human Smad1 polyclonal antibody, 1:400) (Bioss Cor., Beijing; catalog number: bs-1619R) were applied to sections at 4°C and refrigerated overnight. The sections were incubated for 20 minutes at room temperature after addition of polymer helper and incubated for another 30 minutes at room temperature after applying secondary antibodies (Bioss Cor., Beijing; catalog number: bs-0295M-HRP). The reaction product was visualized by incubation with the substrate/chromogen 3,3′-diaminobenzidine (DAB). Finally, the sections were counterstained with hematoxylin (0.02%). Phosphate buffer solution (PBS) was served as the negative control (NC). Each couple of sections, respectively, included one prostate cancer tissue and one adjacent tissue of the same patient. The five couples were randomly selected from five patients. Five random fields were selected from each slice (×400) to record positive cell number under an optical microscope (magnification, ×400; BX51; Olympus Corporation, Japan). The counting field would be selected if more than 100 cells were present therein. Smad1 expressions would be regarded as 1) positive (+) when more than 10% cells were immuno-reactive, and 2) negative (−) when the proportion of immuno-reactive cells was less than 10%. The results of immunohistochemistry were recorded and calculated blindly by two investigators.

### Cell culture

Three kinds of human PCa cell lines [*i.e*. LNCaP, PC-3 and DU145 (ATCC-HTB-81)] and the normal prostate epithelial cell line (*i.e*. RWPE-1) were purchased from Insitute of Biochemistry and Cell Biology (Shanghai). Cells were cultivated in the mixed solution of RPMI-1640 medium (Gibco, USA), 10% newborn bovine serum, 100 IU/mL of penicillin and 100 μg/mL streptomycin within a 5% CO_2_ humidified chamber at 37°C.

### Luciferase reporter assay

Smad1 3′ UTR had two binding sites of miR-199a-3p (Seed1: 5′-ACCTCTGTGACCAAC TATTG-3′; Seed2: 5′-AACTGTATGCTGGCTGTATTACTGT-3′). We constructed mutant Smad1 3′ UTR by site-directed mutagenesis kit (Stratagene, USA) (Seed1 mut: 5′-ACCTCTGTGACCATAATTTG-3′; Seed2 mut: 5′-AACTGTATGCTGGCTGTATATAAGT-3′). Smad1 3′ UTR, Smad1 seed1 mut and Smad1 seed 2 mut were inserted into pRL-CMV vector. After being seeded in a 48-well plate (concentration: 1.0 × 10^4^ per well), PCa cells were co-transfected 24 h later with miR-199a-3p and 1) Smad1 3′ UTR, 2) Smad1 seed1 mut (100 ng), or 3) Smad1 seed2 mut (100 ng), or 4) Smad1 seed1 mut + seed2 mut (100 ng). Lipofectamine 2000 was simultaneously utilized with 1 μL luciferase reporter plasmid and 1 ng pRL-CMV per well (Invitrogen; catalog number: GM021043). On the other hand, the NC group was transfected with empty control (Invitrogen, USA; catalog number: GM-0210NC). Cells were lysed after 48 h of transfection, and luciferase activity was measured using the dual-luciferase reporter assay system according to the manufacturers’ instructions (Promega, USA). Data were normalized by dividing firefly luciferase activity by that of Renilla luciferase for the purpose of transfection efficiency.

### Cell transfection

Cells were divided into four groups of blank, negative control (NC), miR-199a-3p transfection group, and miR-199a-3p + Smad1 transfection group. Transfection was performed by using Lipofectamine 2000 (Invitrogen, USA). Cells in the blank group were not given any treatment. Cells in other groups were transfected with scrambled miRNA mimics (GenePharma Cor., Shanghai; catalog number: E-S-4/5), miR-199a-3p mimics (GenePharma Cor., Shanghai; catalog number: E-S-4/5) and Smad1-complementary DNA (cDNA, Molbase, USA). Complete medium was replaced after 6 h and then cell culture was continued for further experiments.

### MTT cell proliferation assay

When the density of transfected cells reached about 80%, cells were washed for 2 times using PBS. After being digested with trypsin into cell suspension, cells were inoculated into 96-well plates. Each well (volume: 200 μL) included 3 × 10^3^−6 × 10^3^ cells, and a total of 6 wells were replicated. Subsequently, MTT (20 μL, 5 mg/mL, Sigma, USA) was placed into each well and the cells were then cultured in an incubator with 5% CO_2_ at 37°C for 4 h. Dimethyl sulfoxide (DMSO, 150 μL) was added to each well and cells were gently shaken for about 10 minutes in order to dissolve the crystal. A microplate reader was used to detect the absorbance value of each well at a wavelength of 490 nm at the same time of five days. The experiment was repeated for three times.

### Transwell migration and invasion assays

After 72 h of transfection, cells were placed in the upper chamber, and FBS-containing medium was put in the lower chamber as a chemoattractant. Cells were incubated in the 5% CO_2_ chamber at 37°C for 48 h. Non-migratory cells on the upper surface of the membrane were scraped off with cotton swabs. Cells that migrated to the bottom of the membrane were stained for 30 min (Costar Corporation, USA), and the stained cells were viewed under the optical microscope (Nikon, Japan). Five randomly selected fields were quantified using × 200 magnification, and the average number of cells was calculated.

Transwell invasion was performed utilizing 24-well Transwell plates with 8 μm pores, and coating Matrigel (BD Bioscience, US) was introduced to measure the invasive conditions of cells. Briefly, cells were trypsinized and resuspended in a serum-free medium after 48-hour transfection. A total of 2 × 10^4^ cells were added to the upper transwell chamber, and then complete RPMI-1640 medium (volume: 0.5mL) was added into the lower chamber as the chemoattractant. After cells were incubated at 37°C for 24 h, non-invasive cells in the top chamber were removed by cotton swabs. Invasive cells at the bottom of the membrane were fixed with methanol and then stained with crystal violet. The number of invasive cells was finally counted.

### Real time-quantitative polymerase chain reaction (RT-qPCR)

Total RNA extraction was conducted with aid of Trizol reagent kit (Ambion, USA) in line with the manufacturers’ descriptions. After added poly(A) tail on miRNA, the cDNAs were acquired based on oligo(dT)_18_ primers and M-MLV reverse transcriptase utilizing the reverse transcription Kit (Fermentas, Canada). RT-qPCR assay was conducted with qPCR instrument (Fermentas, Canada) to detect relative expression levels of miR-199a-3p and Smad1 mRNA. The primers synthesized by Invitrogen were displayed in Table 1. The reaction conditions were set as follows: 1) pre-degeneration at 95°C for 10 minutes, 2) degeneration at 95°C for 10 seconds, and 3) annealing at 60°C for 20 seconds and extension at 72°C for 35 seconds. The relative expression quantity was calculated using the 2^−△△Ct^ method. The experiment was replicated for three times.

**Table 1:** Primers sequences used for quantitative RT-PCR

Genes	Primer pair sequences
MiR-199a	5′-CAATCGCTTTCAAATAG-3′
	5′-CAGGAGATGCTGTCATC-3′
U6	5′-CTCGCTTCGGCAGCACA-3′
	5′-AACGCTTCACGAATTTGCGT-3′
Smad1	5′-AACCGTTTGAAGTGTAA-3′
	5′-AAACAAACTGGGAAAGA-3′
GAPDH	5′-GTGGACCTGACCTGCGTCT-3′
	5′-GGAGGAGTGGGTGTCGCTGT-3′

### Western blotting assay

Cells were washed with PBS for 3 times and were lysed with M-PER protein extraction reagent with protease inhibitors included (Pierce, USA). Lysates were then collected after centrifugation at 12,000 rpm for 10 min. Protein concentration was determined by a bicinchoninic acid (BCA) protein assay kit. Equal proteins were firstly separated by sodium dodecyl sulfate–polyacrylamide gel electrophoresis (SDS-PAGE), then transferred to equilibrated polyvinylidene difluoride membranes, and finally blocked by 5% non-fat milk Tris-buffered saline (pH 7.4) containing Tween 20 for 2 h. Subsequently, the primary antibody anti-Smad1 (Sino Biological Inc., USA; catalog number: 101176-T08) were incubated over night at 4°C, while secondary antibodies (Abcam, USA; catalog number: ab98624) were incubated for 1 h at room temperature. Ultimately, proteins were visualized using ECL plus Western blotting detection reagents (Amersham Biosciences, USA) and detected with enhanced chemiluminescence (Amersham Pharmacia Biotechnology, USA).

### Statistical analysis

Statistical analyses were analyzed using the GraphPad Prism 5.0 software (GraphPad Software Inc., USA). Counting data was analyzed by chi-square test. Measurement data were showed as mean ± standard deviation (SD). Difference in measurement data between groups was analyzed using the *t*-test or the rank sum test. It would be considered statistically significant when *P* < 0.05.

## Abbreviations

PCa: prostate cancer
BMPs: bone morphogenetic proteins
DAB: diaminobenzidine
PBS: phosphate buffer solution
miRNAs: micro RNAs
MAPKs: Mitogen-activated protein kinases
PBS: phosphate buffer solution
NC: negative control
cDNA: complementary DNA
RT-qPCR: real-time quantitative polymerase chain reaction
TBST: Tris buffered saline with Tween
GRP78: glucose-modulated protein 78
mTOR: mammalian target of rapamycin
TGFβ: transforming growth factor beta
BMP: bone morphogenetic protein
